# Exercise-induced bronchoconstriction: prevalence, pathophysiology, patient impact, diagnosis and management

**DOI:** 10.1038/s41533-018-0098-2

**Published:** 2018-08-14

**Authors:** Bhumika Aggarwal, Aruni Mulgirigama, Norbert Berend

**Affiliations:** 1 Respiratory, Global Classic & Established Products, GSK, Singapore, Singapore; 2 Respiratory, Global Classic & Established Products, GSK, Middlesex, London, UK; 3 Global Respiratory Franchise, GSK, Middlesex, London, UK; 4 George Institute for Global Health, Newtown, NSW Australia

## Abstract

Exercise-induced bronchoconstriction (EIB) can occur in individuals with and without asthma, and is prevalent among athletes of all levels. In patients with asthma, symptoms of EIB significantly increase the proportion reporting feelings of fearfulness, frustration, isolation, depression and embarrassment compared with those without symptoms. EIB can also prevent patients with asthma from participating in exercise and negatively impact their quality of life. Diagnosis of EIB is based on symptoms and spirometry or bronchial provocation tests; owing to low awareness of EIB and lack of simple, standardised diagnostic methods, under-diagnosis and mis-diagnosis of EIB are common. To improve the rates of diagnosis of EIB in primary care, validated and widely accepted symptom-based questionnaires are needed that can accurately replicate the current diagnostic standards (forced expiratory volume in 1 s reductions observed following exercise or bronchoprovocation challenge) in patients with and without asthma. In patients without asthma, EIB can be managed by various non-pharmacological methods and the use of pre-exercise short-acting β_2_-agonists (SABAs). In patients with asthma, EIB is often associated with poor asthma control but can also occur in individuals who have good control when not exercising. Inhaled corticosteroids are recommended when asthma control is suboptimal; however, pre-exercise SABAs are also widely used and are recommended as the first-line therapy. This review describes the burden, key features, diagnosis and current treatment approaches for EIB in patients with and without asthma and serves as a call to action for family physicians to be aware of EIB and consider it as a potential diagnosis.

## Introduction

Exercise-induced bronchoconstriction (EIB) was first recognised as a condition in the 1960s, when it was noted that the forced expiratory volume in 1 s (FEV_1_) in some patients with asthma fell below the resting level during and after exercise compared with other patients with asthma, whose FEV_1_ returned to normal 10–15 min post exercise.^1^ This phenomena was first given the term of exercise-induced asthma (EIA),^2^ subsequently exercise-induced bronchospasm^3^ and finally EIB in 1970.^4^ The introduction of lung function tests, performed before and repeatedly after exercise, helped to identify EIB.^5–7^ Cut-off points were introduced for FEV_1_ (13% reduction) to reduce the likelihood of misclassifying children without EIB.^8^ These methodologies led to the discovery that EIB was affected by environmental factors, such as air temperature and humidity. EIB symptoms were improved by inhaling humid air at ambient temperatures and were completely prevented by inhaling fully saturated air, warmed to body temperature. These experiments formed the basis of the heat vs osmotic hypothesis to describe EIB pathophysiology.^9^ Today, updated international guidelines provide a summary of standard approaches to the diagnosis and management of EIB.^10–12^

EIB mostly presents in patients with asthma, but can also be experienced by individuals without asthma, including athletes.^11,13–16^ The number of patients with EIB is likely to be underestimated, due to the limited number of studies investigating the prevalence of EIB in patients both with and without asthma. This has contributed to a lack of awareness among physicians and the general population.^13^ Access to effective diagnostic methods is limited, resulting in under- or mis-diagnosis.^13^ In addition, there is a risk that physicians will misdiagnose EIB as asthma, and subsequently over- or undertreat the disease. Because EIB can restrict a patients’ ability to exercise and can negatively impact their quality of life (QoL),^14,17^ there is a growing consensus that the management of EIB needs to be improved so that patients with the condition can continue to lead a physically active lifestyle. This review aims to increase awareness of EIB by providing an update on its burden, key features, diagnosis and current treatment approaches.

## Definition and prevalence

EIB is defined as acute airway narrowing (which is transient and reversible) that occurs during or after exercise and can be observed in both patients who have and those who do not have chronic asthma.^11,18^ Typical symptoms include dyspnoea, wheezing, cough, chest tightness, excessive mucus production or the feeling of a lack of fitness when the patient is in good physical condition.^12,13^ EIB reportedly usually occurs within 2−5 min after exercise, peaks after 10 min and resolves in approximately 60 min.

### Prevalence of EIB in the general population

The prevalence of EIB in the general population is approximately 5−20%.^19–23^ However, because few epidemiological studies differentiate people with asthma from the general population, the true prevalence of EIB within the non-asthmatic general population is poorly understood.^12^

The prevalence of EIB is greater in high-performance athletes than in the general population owing to prolonged inhalation of cold, dry air and airborne pollutants.^18^ Studies have reported a prevalence of EIB among elite or Olympic-level athletes of 30–70%,^15,19^ but reports are variable depending upon the environment in which the sport is performed, the type of sport and the maximum intensity achieved.^12^

**Fig. 1:**
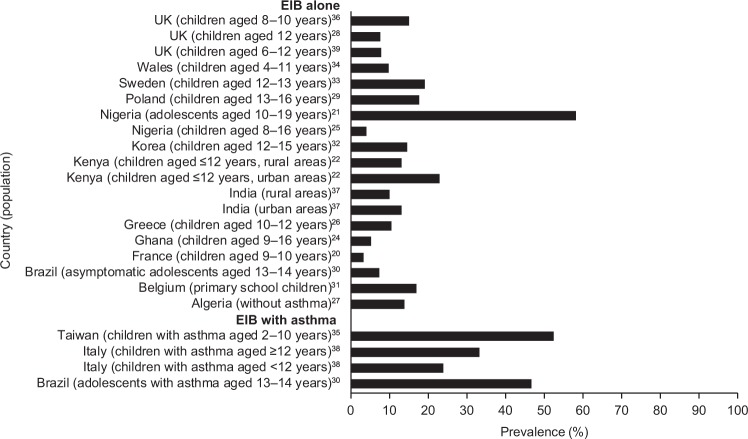
Country-specific prevalence* of EIB in children (general population).^20–22,24–39^ *Owing to differences in study methodology, comparisons between studies should be treated with caution. EIB exercise-induced bronchoconstriction

In children, the prevalence of EIB is also higher than in the general population, ranging from 3 to 35% (children ≤16 years old) (Fig. 1).^20–22,24–39^ There is large variation in the prevalence of EIB in children worldwide, with studies conducted in Nigeria,^25^ Brazil^30^ and Poland^29^ reporting higher rates of EIB than Ghana,^24^ India^40^ and Greece^26^ (Fig. 1). The impact of ethnicity on the prevalence of EIB is unclear, as only one study has directly compared prevalence between different ethnic groups in Scottish and English children.^41^ Children from an Asian background were 3.6 times more likely to experience EIB compared with Caucasian inner-city children.^41^ The prevalence of EIB was 12.3% in children with Asian ethnicity compared with 9.1% in Afro-Caribbean children and 4.5% in Caucasian inner-city children.^41^ These results should be interpreted with care because studies of ethnicity are invariably confounded by non-genetic factors.

Children living in urban environments are 1.6 times more likely to experience EIB compared with those living in rural areas, based on a study conducted in Kenya.^22^ The higher rates observed in urban areas were partially explained by an increased family history of asthma symptoms or increased exposure to environmental factors in urban areas, such as vehicle fumes, crowdedness and household animals.^22^ Similar urban–rural difference were observed in India. In addition, children with a low or middle socio-economic status were 8–10% more likely to experience EIB than those with a high socio-economic status.^25^ However, this finding is not universal; a study conducted in Nigeria demonstrated that EIB was not related to socio-economic class.

### Prevalence of EIB in patients with asthma

Asthma is the main co-morbid factor associated with EIB, and EIB is estimated to occur in approximately 90% of patients with asthma.^12,19^ Patients with poorly controlled or severe asthma are more likely to manifest with EIB than patients with well-controlled or milder disease.^12,19^ Consequently, between-country differences in the prevalence of EIB should be considered in the context country-specific asthma control levels.^42^ In children and adolescents with asthma, the prevalence of EIB is estimated to be approximately 20–90%,^29,30,35,38^ with one study reporting that 46.7% of children with asthma display symptoms of EIB compared with 7.4% of those who do not have asthma.^30^ The majority of patients with chronic asthma will likely experience a transient increase in symptoms following an appropriate exercise challenge. EIB is known to hinder children’s participation in vigorous activities. Other risk factors contributing to the prevalence of EIB include allergic rhinitis, a personal history of allergies, history of asthma in a close relative or history of wheeze.^20,21,30,35^

### Challenges of determining EIB prevalence and future work

It remains challenging to understand the extent of EIB within the general non-asthmatic population and among patients with asthma when such substantial variability in the prevalence of EIB is reported. This variability is likely due to differences in geographical regions and population characteristics (age, background, diagnosis of asthma) and differences in study design. The prevalence of EIB may be affected by the type of exercise test used to induce symptoms (treadmill, cycling, free running) or the diagnostic method used to define EIB (FEV_1_, peak expiratory flow (PEF), direct/indirect bronchial provocation tests or self-reported).^19,20,22^ Moreover, the lung function index used (time of pre- and post-exercise measurements), temperature, seasons and humidity are also factors that may have affected prevalence data.^19,20,22^ The influence of these factors highlights the need for standardised diagnostic measures to more accurately assess the prevalence of EIB. In addition, there is a pressing need for more epidemiological studies to assess the prevalence of EIB in the general population, excluding patients with asthma, to allow the prevalence of EIB without asthma to be better understood.

## Pathophysiology

At present, the osmotic theory is widely accepted as the established underlying mechanism of EIB. The osmotic theory suggests that increased ventilation in the airways during periods of exercise leads to water loss from the airway surfaces by evaporation, thus dehydrating the airway surfaces and initiating the events that lead to the contraction of bronchial smooth muscle.^43^ During exercise-related hyperventilation, transient osmotic change at the airway surface occurs owing to reductions in epithelium liquid volume, which in turn triggers mast cell degranulation.^43^ Consequently, there is mast cell-mediated release of prostaglandins (prostaglandin D2), leukotrienes, histamine and tryptase. These signalling molecules are known to mediate airway smooth muscle contraction and increase mucus production and microvascular permeability and sensory nerve activation, and their release is thought to be the main stimulus for bronchoconstriction and airway oedema.^43^

### Precipitating factors for EIB

In patients with EIB and chronic asthma, the pathophysiological mechanisms described above simply represent a trigger of underlying airway hyperactivity associated with poorly controlled asthma.^44^

On the other hand, in patients with EIB who do not have asthma, the mechanisms described by the osmotic theory are believed to be directly responsible for causing bronchoconstriction and associated symptoms. Intense ventilation of cold air can further increase dehydration of the airway surfaces and cause changes in bronchial blood flow, explaining why athletes performing in cold weather (e.g., ice hockey, Nordic skiing) demonstrate the highest rates of EIB.^10,45^ Epithelial injury that is caused by the inhalation of air pollutants and poorly conditioned air during exercise has also been hypothesised to be a contributing factor for the development of EIB in patients without asthma.^46,47^ This hypothesis likely explains why reported prevalence rates for EIB in competitive swimmers approach 50%, with exposure to chloramines from the pool water considered the probable cause of epithelial injury.^45^ Supporting this theory, a family or personal history of atopy to environmental factors has been identified as a known risk for EIB.^45^

## Impact of EIB on patients

EIB is associated with both a physical and an emotional burden. From our review of the literature, we found that a limited number of studies have investigated the emotional burden associated with EIB. A large-scale, survey-based study of more than 30,000 children aged 6–14 years in Japan revealed that children self-reporting symptoms of EIB with or without asthma had significantly lower QoL scores than children without EIB (*p* < 0.001).^48^ For children with asthma, the presence of EIB had a significant negative association with QoL regardless of the severity of asthma symptoms.^48^ In the United States, adolescent athletes with or without asthma who reported dyspnoea during exercise (*n* = 32) showed significantly lower scores for health-related QoL (HRQoL), including sub-scores for physical functioning, general well-being and emotional functioning, than those without exercise-associated dyspnoea (*n* = 128).^49^ However, adolescents with spirometry-defined EIB compared with non-spirometry-defined EIB in this study did not show significant reductions in HRQoL, possibly owing to the low number of patients included (*n* = 18).^49^ A similar Swedish study of adolescents with or without asthma (*n* = 140) demonstrated a significant association between spirometry-defined EIB and reduced HRQoL.^50^ Interestingly, this effect was revealed to be primarily driven by reduced total HRQoL and physical function in girls with EIB, with no significant difference evident between boys with or without EIB.^50^ Girls with EIB also exhibited significantly higher scores for anxiety, but not depression, compared with girls without EIB.^50^ A telephone-based survey, the Exercise-Induced Bronchospasm Landmark National Survey in the United States, provided comprehensive information relating to exercise-induced respiratory symptoms from the perspective of both the general population (*n* = 1085) and adults with EIB and asthma (defined as those who reported taking asthma medication in the previous year; *n* = 1001).^14^ The survey found a significant burden of disease associated with EIB, including emotional burden.^14^ Patients with asthma who reported ≥1 symptom of EIB reported feeling more fearful (10.9 vs 27.7%; *p* < 0.001), isolated (6.0 vs 15.1%; *p* < 0.01), depressed (9.1 vs 23.4%; *p* < 0.001), frustrated (22.9 vs 54.5%; *p* < 0.001) and embarrassed (4.2 vs 20.0%; *p* < 0.001) compared with those not reporting EIB symptoms.^14^ While current evidence indicates a significant functional and emotional impairment among patients with EIB and asthma, there is a need for more studies to assess the burden of disease and HRQoL among patients with objectively measured EIB and underlying asthma, as well as among patients with EIB alone.

Almost half of patients (45.6%) with asthma reported impact on both their participation and performance in sports, and a similar number (42.7%) reported they could not keep pace with peers during physical activities.^14^ A systematic review of studies assessing the impact of EIB on athletic performance failed to show a significant effect but did highlight the need for more well-designed, sport-specific studies on the physiological impact of EIB.^51^

## Impact of effective EIB management

**Fig. 2:**
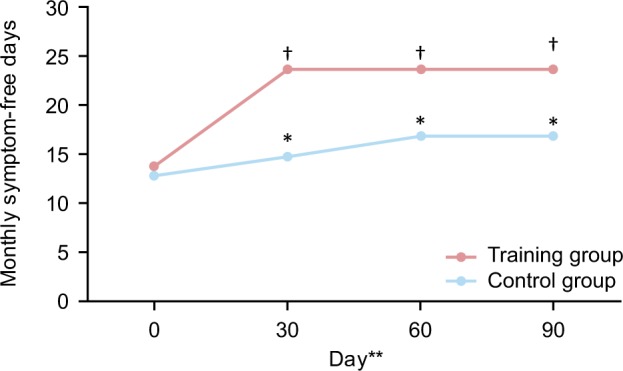
Impact of aerobic training on symptomatic burden in patients with moderate or severe persistent asthma.^54^ Patients were 20–50 years old with moderate or severe persistent asthma. Patients were under medical treatment for 6 months and considered clinically stable; *p < 0.05 compared with baseline; ^†^*p* < 0.05 compared with baseline and control group (two-way repeated-measure analysis of variance). Control group, *n* = 45; aerobic training group *n* = 44. **Time points are 0 days (1 month before treatment), 30 days (first month of treatment), 60 days (second month of treatment) and 90 days (third month of treatment)^54^

Given the well-known health benefits of exercise in both the general population and individuals with asthma,^52^ the need to manage EIB effectively is clear. Exercise, in particular swimming,^53^ has been shown to improve lung function and asthma symptoms and outcomes, including QoL in patients with asthma.^54^ An analysis of the impact of an aerobic training programme (*N* = 101) on asthma-specific health-related QoL, asthma symptoms, anxiety and depression scores in patients with moderate or severe persistent asthma found that aerobic training had an important role in the clinical management of persistent asthma.^54^ Significant (*p* < 0.001) reductions in physical limitation and symptom frequency (Fig.2) were reported in the training group compared with the control group. Moreover, only patients from the training group reported reductions in anxiety and depression levels (*p* < 0.001).^54^

Many patients stop exercising because of their EIB symptoms. In the 2011 EIB Landmark Survey, 22.2% of children with asthma aged 4−12 years and 31.8% of those aged 13−17 years avoided sports activities as a result of their EIB. As EIB affects up to 90% of patients with asthma,^12^ the potential impact on aerobic exercise participation is substantial. Arguably, patients with asthma and EIB are at greater disadvantage than those with asthma and no EIB, for symptom precipitation during exercise often leads to avoidance of regular exercise and reduced QoL. It is important to raise awareness in primary care settings that EIB restricts exercise in patients with asthma, given the clinical and psychosocial benefits associated with physical activity.

## Diagnosis

The diagnosis of EIB in patients with and without asthma is multifactorial, leading to the condition often being either under- or over-diagnosed.^13^ A recent systematic review found insufficient evidence to support the widespread adoption of any existing EIB screening tools, and highlighted that there exists a substantial unmet need for a validated questionnaire.^13^ Here we will discuss a number of diagnostic methods that are currently used for diagnosing EIB in both patients with underlying asthma and in those with EIB alone.

**Fig. 3:**
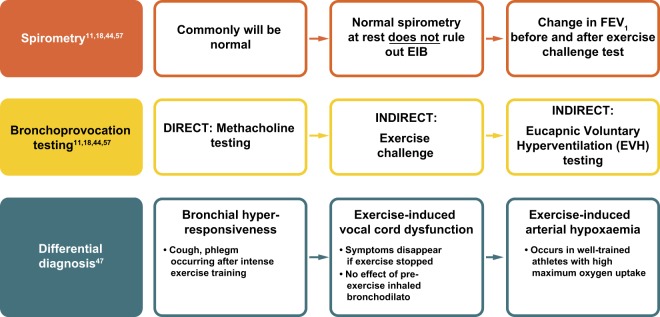
Algorithm for diagnosis of EIB.^11,18,44,47,57^ EIB exercise-induced bronchoconstriction, FEV_1_ forced expiratory volume in 1 s

EIB should be considered when patients report respiratory symptoms that are induced by exercise. One potential approach for family physicians is to ask the patient to measure his/her PEF after the typical exercise that usually provokes symptoms.^55,56^ If peak flow results are reduced compared with the patient’s baseline readings, formal investigation is required. Diagnosis of EIB is confirmed based on specific changes in lung function provoked by exercise, rather than on the basis of symptoms.^11,18^ Such testing can involve the use of both spirometric and bronchoprovocation techniques (Fig. 3; see refs. ^11,18,44,47,57^).^43^

### Spirometry

The American Thoracic Society (ATS) Clinical Practice Guidelines outline a decline in FEV_1_ of ≥10% from baseline after exercise or hyperpnoea challenge as confirmation of a positive EIB diagnosis.^11^ A minimum of two reproducible FEV_1_ measurements are taken in series post-exercise challenge, with the highest acceptable value being recorded at each interval (usually 5, 10, 15 and 30 min after exercise). The lowest percentage decline in FEV_1_ within 30 min post exercise from the pre-exercise level can then be used to determine the severity of EIB (mild, 10– < 25%; moderate, 25– < 50%; severe ≥50%).^11^

### Bronchoprovocation testing

Many protocols recommend breathing dry air (10 mg H_2_O/L) with a nose clip in place while completing an exercise challenge. Several surrogates for exercise testing in the form of bronchoprovocation tests are available which, depending upon available resources, may be more suitable than a dry air exercise challenge.^11^ The widely used methacholine challenge is a direct bronchoprovocation test; two versions of the methacholine challenge are used, a standard protocol recommended in ATS guidelines, and a second, more rapid protocol.^58,59^ Alternatively, there are a number of indirect bronchoprovocation tests. The Eucapnic Voluntary Hyperventilation (EVH) test was developed specifically for identifying EIB.^60^ Dry air (containing 5% carbon dioxide) is hyperventilated at room temperature for 6 min at a target ventilation of 30 times the subject’s FEV_1_, with a reduction of ≥10% of the pre-test value being diagnostic of EIB.^60^ EVH testing is considered a reproducible, well-standardised test that is both quick and easy to administer; however, it is laboratory dependent and thus not widely available.^13^ Other indirect bronchoprovocation tests include the hypertonic saline challenge and the mannitol test.^61^ The latter was developed to improve the availability and standardisation of osmotic challenge testing;^62,63^ however, the sensitivity and specificity of the mannitol challenge has yet to be well established.^11,13^

While none of these bronchoprovocation tests are sensitive or specific to EIB, they all complement clinical history to identify airway hyperresponsiveness consistent with a diagnosis of EIB.^11^ In addition, although these tests may be used for diagnosis of EIB in patients with and without underlying asthma, it has been suggested that indirect bronchoprovocation tests better reproduce the effects of exercise and may therefore be more accurate in diagnosing EIB in patients without asthma.^44^

### Distinguishing EIB from asthma

A key consideration for physicians when a patient presents with symptoms of wheeze and shortness of breath triggered by exercise is whether a diagnosis of asthma with EIB or EIB alone is appropriate. The management of EIB in patients without asthma is very different from the management of patients who experience EIB in association with poorly controlled asthma. As such, it is crucial to avoid over-diagnosis of asthma and subsequent over- or under-treatment.

The Global Initiative for Asthma (GINA) Guidelines outline several symptoms that increase or decrease the probability of a patient having asthma.^52^ Most notably, symptoms that often worsen at night or in the early morning, that vary over time and in intensity, and that are triggered by exercise, viral infections, irritants and allergens increase the probability of asthma. Conversely, exercise-induced dyspnoea with noisy inspiration decreases the probability of asthma.^52^ The guidelines also highlight the importance of determining if the patient’s symptoms occur only during or after exercise, and if the patient has any other risk factors for exacerbations. If symptoms are solely related to exercise, and there is no additional risk of exacerbation, a diagnosis of EIB rather than asthma should be considered.^52^

### Differential diagnosis

In the absence of airway hyperresponsiveness to challenge, differential diagnoses must be considered, particularly in adolescent athletes. Consideration must be given to the following conditions: bronchial hyperresponsiveness (the occurrence of cough or phlegm after intense exercise); exercise-induced vocal cord dysfunction (symptoms disappear when exercise is stopped and there is no observed effect of pre-exercise inhaled bronchodilator); and exercise-induced arterial hypoxaemia (occurring typically in well-trained athletes with high maximum oxygen uptake).

A Joint Task Force for defining practice parameters for the management of EIB (2016) suggested physicians can also consider cardiopulmonary exercise testing to determine if symptoms are resulting from exercise-induced dyspnoea and hyperventilation, particularly in children and adolescents.^43^ Shortness of breath during exercise can also be associated with underlying conditions such as chronic obstructive pulmonary disease or restrictive lung conditions (e.g., obesity).^43^ A history of shortness of breath alongside other systemic symptoms (e.g., pruritus, urticaria and hypotension) may rarely be indicative of exercise-induced anaphylaxis.^43^ Finally, if EIB has been ruled out, referral to a specialist should be considered for patients who present as breathless when exercising (with or without chest pain) and for whom heart disease or other conditions are suspected.^43^

## Under-diagnosis and under-treatment

There is growing evidence that objectively confirmed EIB is more prevalent than would be assumed from using self-reported symptoms alone,^15,64^ possibly because a decline in lung function post exercise (the criterion for EIB) may occur in the absence of symptoms.^23^

A prospective study of varsity-level college athletes in the United States found that the use of symptoms to diagnose EIB is not predictive of whether athletes have objectively documented EIB. Of the 107 athletes included in the study, 42 (39%) recorded EVH results considered positive for EIB.^15^ Of these athletes, 86% (36/42) reported no previous history of asthma. The EVH-confirmed prevalence of EIB was 36% in athletes without EIB symptoms compared with 35% in those with EIB symptoms. As such, the authors concluded that the empiric diagnosis and treatment of EIB following self-reported symptoms alone may result in an increase in inaccurate diagnoses and ultimately increased morbidity.^15^ These results are corroborated by a study of elite British athletes, which showed that the majority (73%) with EVH-confirmed EIB were previously undiagnosed.^16^

Failure to adequately diagnose EIB is also likely to result in under-treatment of symptoms. A survey conducted solely among patients with asthma found that although 83% of participants with asthma experienced at least one exercise-related respiratory symptom (shortness of breath, wheezing, coughing, difficulty taking a deep breath, noisy breathing or chest tightness during or immediately after exercising), only 30.6% reported a diagnosis of EIB. Importantly, despite these impairments, few respondents adhered to treatment guidelines relating to prophylactic medication prior to exercise.^14^

Overall, current estimates reveal that approximately 70% of patients with asthma and EIB are diagnosed based on history and symptoms alone, and only 18% following exercise, medication or lung function testing (Table 1^65^). A survey indicates that family physicians, in particular, are significantly less likely than pulmonologists to utilise objective testing for EIB.^66^ This is likely to be due, at least in part, to access issues. Among family practitioners in England, 85% reported that they had no access to bronchoprovocation testing; 11% had access to laboratory-based exercise testing; and 4% had access to EVH, methacholine or mannitol provocation testing.^67^

**Table 1:** The basis of diagnosis of EIB^65^

	Percentage of patients
History and symptoms alone	70
Exercise test	10
Medication test	3
Lung function test/spirometry	5
Other	2
Not sure	10

*EIB* exercise-induced bronchoconstriction

## Treatment of EIB

### Treatment of EIB in patients without asthma

For patients without underlying asthma, management of EIB should focus on relief of bronchoconstriction, and the reduction in risk (or prevention entirely) of the occurrence of bronchoconstriction, to allow the patient to continue to engage in physical exercise with minimal respiratory symptoms. There are many non-pharmacological approaches recommended to reduce the risk of bronchoconstriction, which include warm-up before exercise to induce a refractory period; interventions that pre-warm and humidify inhaled air during exercise (e.g., breathing through a face mask or scarf) and avoiding high exposure to air pollutants and allergens.^11,44^ Some athletes use a physical warm-up of 10–15 min of moderately vigorous exercise before the planned period of exercise or competition to induce a so-called 'refractory period', during which EIB symptoms may be reduced.^43^ If EIB symptoms continue despite these non-pharmacological approaches, use of pharmacological methods such as short-acting β_2_-agonists (SABAs) 15 min before exercise, leukotriene receptor antagonists (LTRAs) or chromones should be considered as alternative pre-exercise treatments in accordance with guidelines recommendations.^52^

### Treatment of EIB in patients with asthma

EIB in patients with asthma can be a sign of poor asthma control. In these cases, management of EIB should focus on following global treatment guidelines to ensure the underlying asthma is controlled.^52^ Those patients who achieve good overall asthma control but retain EIB will require additional treatment. In addition to the non-pharmacological approaches described above,^11^ guidelines recommend various pharmacological therapies to help prevent EIB in patients with chronic asthma.

Currently, patient understanding of EIB treatment may be characterised as inadequate. Only 22.2% of individuals experiencing exercise-related symptoms reported taking quick-relief medications prior to exercise ‘always’ or ‘most of the time’ (with this proportion increasing to just 38% in cases of diagnosed EIB).

The authors of the EIB Landmark Survey concluded that their findings highlighted an urgent need for better asthma education, with almost one-third of people with asthma reporting that they take rescue medication ≥3–6 times per week for uncontrolled asthma symptoms.^14^ They suggest that exercise-related symptoms in this population reflect inadequate management of the underlying disease. Notably, 37% of patients with asthma were unaware that exercise-related symptoms indicate poor asthma control.^14^ This finding highlights the need to confirm or refute a diagnosis of asthma as the first step in EIB management.

### The ATS guidelines

The ATS guidelines^11^ acknowledge that EIB may be present in both patients with and without asthma, and as such do not make specific recommendations based on the presence of asthma.

In patients diagnosed with EIB and asthma, the use of an inhaled SABA, typically 15 min before exercise, is strongly recommended^11^ However, daily use of SABAs has been shown to lead to tolerance, and therefore should be used to prevent EIB on an intermittent basis only (i.e., less than daily on average).^11^

Although not licensed specifically for EIB, the ATS recommends daily use of inhaled corticosteroids (ICS) for these patients, though it recognises that maximal improvement may require 2–4 weeks of treatment. The main benefit of ICS is as maintenance therapy to address underlying suboptimal control of asthma symptoms. The ATS recommendation against the use of a single dose of ICS immediately before exercise reflects this understanding. For patients who continue to have symptoms despite using an inhaled SABA before exercise, or who require an inhaled SABA daily or more frequently, daily use of long-acting β_2_-agonist (LABA) as a single therapy is not recommended due to known associations with acute exacerbations.^68,69^ When EIB is unresponsive to SABA therapy, daily use of an LTRA taken at least 2 h before exercise or pre-exercise use of a mast cell stabiliser are recommended.

#### Guidelines on EIB by the Joint Task Force on Practice Parameters

This practice parameter summary is a 2016 update of contemporary practice guidelines first published in 2010 and based on a systematic literature review.^12,43^ The updated guidelines recommend the use of SABAs for protection against EIB in both patients with and without asthma, and for accelerating recovery of pulmonary function. The Task Force recommends caution regarding the daily use of SABA alone or in combination with ICS for the management of EIB owing to the potential for tolerance (leading to a reduced duration/magnitude of effect). ICSs in combination with other preventive therapies are considered a good treatment option because of their ability to decrease the frequency and severity of EIB, although they do not necessarily eliminate it in patients with asthma. However, the guidelines do note that the use of ICS in the prevention of EIB in patients without asthma is controversial owing to a current lack of support from ad hoc designed clinical trials and impaired responses in patients with underlying neutrophilic inflammation. Consistent with the ATS guidelines, the use of daily LABAs with ICS therapy is not recommended for EIB unless this approach is needed to treat underlying moderate to severe persistent asthma.

Both LTRAs and mast cell-stabilising agents are considered suitable pre-exercise treatment options.^43^ Inhaled ipratropium bromide should be considered for patients who have not responded to other agents; however, its ability to attenuate EIB is considered inconsistent.^43^

### Recommended treatment options: the evidence

#### Short-acting β_2_-agonists

**Fig. 4:**
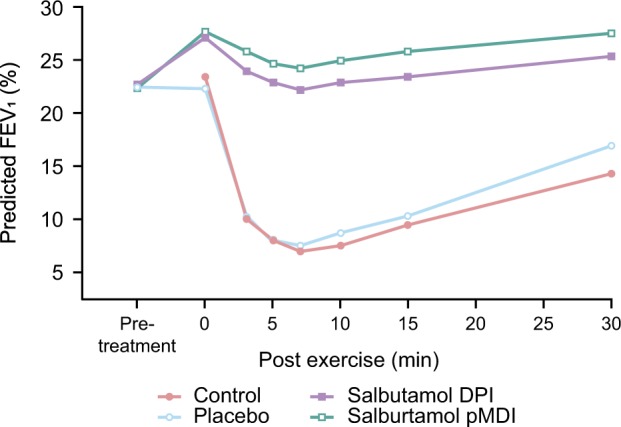
Mean values for forced expiratory volume in 1 s in patients with exercise-induced asthma treated with a short-acting β_2_-agonist.^70^ Data are expressed as a percentage of the predicted normal value, measured before and 30 min after each treatment and for 15 min after exercise. Crossover study conducted in 27 patients. Reproduced from Anderson et al. (2001) with permission from Wolters Kluwer Health, Inc. DPI dry powder inhaler, FEV_1_ forced expiratory volume in 1 s, pDMI pressurised metered dose

SABAs are the single most effective therapeutic agents for the acute prevention of intermittent EIB^43^ (Fig. 4). SABAs stimulate β_2_-receptors on the surface of the airway smooth muscle, causing relaxation and bronchodilation, as well as possibly preventing mast cell degranulation.^11^ In patients' asthma and EIB, SABAs have been shown to be effective in preventing a fall in FEV_1_ (Fig. 4).^70^ Evidence shows that when combined with pre-exercise warm-ups, SABAs still provide an additive protective effect in patients with asthma and EIB^11^ and confer a greater protective effect against developing EIB than either warm-up or SABA alone.^71^

#### Inhaled corticosteroids

A Cochrane review of results from eight randomised controlled trials involving 162 participants found that ICS taken for 4 weeks pre-exercise can reduce post-exercise declines in FEV_1_ in both children and adults.^72^ ICS is licensed only for patients with asthma and may not be as effective against EIB alone. One study noted that EIB symptoms were unchanged in the majority (67%) of patients with EIB alone following a mean of 22 weeks of ICS therapy.^48^

#### Long-acting β_2_-agonists

The LABA formoterol has also been shown to provide improvements in EIB and but daily use of LABAs with ICS therapy is not recommended for EIB unless to treat underlying moderate to severe persistent asthma.^12,43,11^ Comparisons of SABAs and LABAs (salmeterol) in patients (*N* = 12) with mild-to-moderate stable asthma showed that both treatments reduced mean declines in FEV_1_ following exercise, with the SABA (3.8 ± 5.5%) and LABA (0.83 ± 6.2%) showing large effects 1 h post challenge compared with placebo (27.1 ± 7.3%).^73^ However, a meta-analysis has demonstrated that the bronchoprotective effect of salmeterol at 9 h post treatment is reduced after 4 weeks.^74^

#### Leukotriene receptor antagonists

Finally, LTRAs have also been shown to be efficacious for EIB in patients with and without asthma; LTRAs are also specifically indicated for prophylaxis in patients with asthma and EIB.^75^ Clinical data have shown that once-daily treatment with montelukast (5 or 10 mg tablet) can improve a number of post-exercise deficits in lung function within 3 days in some patients.^76^ In a pooled analysis of seven trials, patients with asthma and EIB had a mean maximum decline in post-exercise FEV1 that was 10.7% less with LTRAs compared with placebo.^11^

### Treatment of EIB in athletes

The treatment of EIB in elite athletes is a topic of particular interest and one that falls outside the scope of this review. Diagnosis and treatment of EIB in elite athletes has been extensively covered by a Joint Task Force Report prepared by the European Respiratory Society, the European Academy of Allergy and Clinical Immunology and GALEN,^10^ as well as the World-Anti-Doping Agency.^77^ Notably, the International Olympic Committee recommend that treatment follows international guidelines as described above; ICS and some inhaled SABAs can be used in accordance with the Therapeutic Use Exemption Standard.^78^ In addition, athletes should be warned of the diminishing therapeutic effects of inhaled SABAs when used frequently, and offered education in order to develop self-management skills and ensure appropriate use of medication.

## Conclusions

EIB can occur in both patients with and without asthma, with the prevalence in patients with asthma estimated at approximately 90%.^12^ EIB may lead to a substantial emotional burden on patients, and restrict exercise and sports participation. This potentially leads to long-term QoL and physical health consequences in patients with EIB, with or without asthma. Increased awareness among patients and physicians of the symptoms and risk factors for EIB and increased use of objective diagnostic tests is key to the holistic management of patients with EIB. As such, there is a pressing need for more research into EIB in patients with and without asthma, and the development of validated and widely acceptable screening methods and/or accurate diagnostic methods, which can be made accessible to family physicians.

For patients with and without asthma, pre-exercise SABAs are recommended as the first-line option for pharmacological treatment of EIB.^11,43^ The primary focus should be to increase awareness of EIB and educate patients to recognise symptoms and risk factors of EIB. Improved diagnosis and patient education further helps to optimise symptom control. Furthermore, increasing the accuracy of EIB diagnoses and providing education in how the patient can use SABA to prevent symptoms are needed.
